# Determining efficient helical IMRT modulation factor from the MLC leaf‐open time distribution on precision treatment planning system

**DOI:** 10.1002/acm2.12581

**Published:** 2019-04-08

**Authors:** Robert Boyd, Kyoungkeun Jeong, Wolfgang A. Tomé

**Affiliations:** ^1^ Department of Radiation Oncology Montefiore Medical Center Albert Einstein College of Medicine Bronx NY USA

**Keywords:** leaf‐open time, modulation factor, tomotherapy, treatment planning

## Abstract

**Purpose:**

Since the modulation factor (MF) impacts both plan quality and delivery efficiency in tomotherapy Intensity Modulated Radiation Therapy (IMRT) treatment planning, the purpose of this study was to demonstrate a technique in determining an efficient MF from the Multileaf Collimator (MLC) leaf‐open time (LOT) distribution of a tomotherapy treatment delivery plan.

**Methods:**

Eight clinical plans of varying complexity were optimized with the highest allowed MF on the Accuracy Precision treatment planning system. Using a central limit theorem argument a range of reduced MFs were then determined from the first two moments of the LOT distribution. A step down approach was used to calculate the reduced‐MF plans and plan comparison tools available on the Precision treatment planning system were used to evaluate dose differences with the reference plan.

**Results:**

A reduced‐MF plan that balanced delivery time and dosimetric quality was found from the set of five MFs determined from the LOT distribution of the reference plan. The reduced‐MF plan showed good agreement with the reference plan (target and critical organ dose‐volume region of interest dose differences were within 1% and 2% of prescription dose, respectively).

**Discussion:**

Plan evaluation and acceptance criteria can vary depending on individual clinical expectations and dosimetric quality trade‐offs. With the scheme presented in this paper a planner should be able to efficiently generate a high‐quality plan with efficient delivery time without knowing a good MF beforehand.

**Conclusion:**

A methodology for deriving a reduced MF from the LOT distribution of a high MF treatment plan using the central limit theorem has been presented. A scheme for finding a reduced MF from a set of MFs that results in a plan balanced in both dosimetric quality and treatment delivery efficiency has also been presented.

## 1 INTRODUCTION

Helical tomotherapy (HT) delivers radiation therapy through synchronization of the binary Multileaf Collimator (MLC) leaf‐pair openings, gantry rotation period, and couch longitudinal speed. Highly conformal dose distributions can be achieved through intensity modulation of the HT radiation field. The HT radiation field is divided into 51 projections per gantry rotation (7.06° of gantry rotation per projection). Each projection is further divided into 64 beamlets representing each of the 64 MLC binary leafs (i.e., the leaf being either open or closed). The leaf‐open time (LOT) of each beamlet that intersects a target volume determines the instantaneous radiation dose delivered from it through the projection arc or fraction thereof. Intensity‐modulated radiation therapy (IMRT) is achieved by varying the LOT of each beamlet with inverse‐planning optimization of the treatment plan.

Inverse‐planning optimization for Tomotherapy requires the planner to choose a modulation factor (MF) that is defined as

(1) $$MF=\frac{\mathrm{LO}T_{\max}}{\mathrm{LO}T_{\mathrm{mean}}},$$

where LOT_max_ is the maximum LOT and LOT_mean_ is the average of all beamlet LOTs.^1^ The MF is a parameter that influences the complexity of intensity‐modulated radiation field and a higher MF may result in a more conformal, homogeneous target dose distribution and improved sparing of critical structures. High MF values are typically used for plans with irregular‐shaped planning target volumes (PTV) and critical structures that are either adjacent or overlapping.^1^ A high MF allows greater freedom for the optimizer to vary LOTs for beamlets.

Modulation factor (MF) values reported in literature vary with institution, treatment site, and type of study. MFs reported for several prostate studies range from 1.8 to 3.5 with 2.5 being the most common factor.^2^, ^3^, ^4^, ^5^, ^6^ MFs reported for several head‐and‐neck (HN) studies range from 2.0 to 3.5.^7^, ^8^, ^9^, ^10^, ^11^ MFs for several gynecological (GYN) studies ranged from 3.0 to 4.0.^12^, ^13^ Most recently Shimizu et al. retrospectively analyzed the MF used in 293 HN plans and 181 prostate plans to derive an initial MF (2.1 and 1.8, respectively) and upper limit MF (2.6 and 2.2, respectively) specific to the treatment site.^14^

The MF has a direct impact on treatment delivery time. Because the linear accelerator dose rate, couch speed, and gantry period are constant during helical treatment delivery, the total time for “beam‐on” delivery is a product of number of gantry rotations and gantry period,

(2) Totaldeliverytime=activegantryrotations×gantryperiod.

The number of gantry rotations is determined by the pitch and the length of cranial‐caudal treatment volume plus jaw width. The gantry period is equal to *51 × *LOT_max_, unless LOT_max_ is <235 ms, in which case the gantry period minimum has been reached at 11.8 s. Therefore*, for gantry periods above 11.8 s*,

(3) $$Total\;delivery\;time=51\times \mathrm{LO}T_{\max}\times \mathrm{active}\;\mathrm{gantry}\;\mathrm{rotations}.$$

A high MF value can allow the optimizer to generate beamlets with long LOTs that have minimal impact on the dose distribution.^15^ It has been suggested for complex plans that planners start with a high MF to achieve a good conformal plan and then reduce the MF until the dosimetric qualities of the plan degrade to clinically unacceptable values.^9^, ^16^

This paper presents a heuristic approach for determining a MF from the first two moments of the LOT distribution of a plan optimized with the highest allowed MF. A technique is then used to determine a set of MFs for subsequent “reduced‐MF” plan calculations to find a balance between dosimetric quality and treatment delivery time.

## 2 MATERIALS AND METHODS

**Figure 1:**
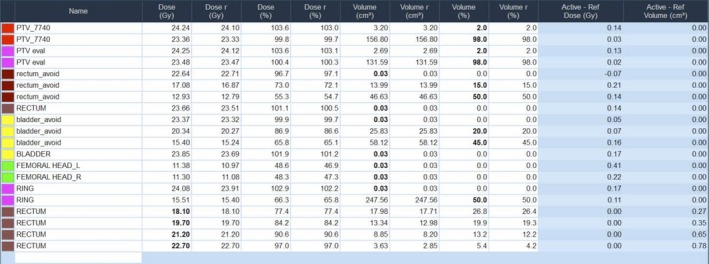
Precision treatment planning system “Dx Vx Value” table for a prostate plan. The planner specifies dose (Gy or % of Rx dose) or volume (absolute or %), shown in bold, and the table shows rest of the information. The last two columns show the difference between current plan and a reference plan in either dose or % volume change.

Table 1 lists the six treatment cases of varying complexity that were used to demonstrate the proof of concept for determining the MF from the moments of the LOT distribution. These plans were taken from actual treatments with same planning structure sets and jaw widths as the original plans. In our clinic we typically use 2.5‐cm jaw width for prostate and HN patients and 5.0‐cm jaw widths for GYN cases with para‐aortic nodal involvement. A pitch value of 0.43, determined from the 0.86/n formula by Kisseck et al, was used for all plans.^17^ No significant dose distribution threading effects were seen with the plans. All treatments utilized dynamic jaws.^18^ Plans were generated with the Precision treatment planning system (TPS) version 1.0.0.2 (Accuray, Sunnyvale, CA) using VoLO, a GPU‐based optimizer incorporating a non‐voxel based algorithm.^19^ VoLO has been shown to produce dosimetrically equivalent plans compared to the original voxel‐based optimization algorithm in a fraction of the computation time.^20^ Although this approach for determining the MF is valid for the original Tomotherapy TPS, the Precision TPS is ideal for this process in that (a) the moments of the LOT distribution are displayed with the distribution in the graphics user interface and (b) two plans can easily be compared with each other with the Precision plan evaluation feature. Figure 1 shows the Precision TPS “Dx Vx Value” table that allows a user to observe specific dose‐volume values of a plan and comparative differences with a reference plan.

**Table 1:** List of clinical test plans

Case	Site	Rx target dose	Description
1	Prostate	23.4 Gy 1.8 Gy/fraction	Two boost plans with different levels of rectal sparing (1.a normal sparing and 1.b aggressive sparing)
2	Prostate	23.4 Gy 1.8 Gy/fraction	Two boost plans with different levels of rectal sparing (2.a normal sparing and 2.b aggressive sparing)
3	GYN	50 Gy 2.0 Gy/fraction	SIB cervical squamous cell carcinoma plan with 45 Gy (1.8 Gy/fraction) to lymph nodes, para‐aortic involvement
4	GYN	50 Gy 2.0 Gy/fraction	SIB cervical squamous cell carcinoma plan with 45 Gy (1.8 Gy/fraction) to lymph nodes, para‐aortic involvement. Kidneys received as low as reasonably allowed (ALARA) dose
5	HN	69.96 Gy 2.12 Gy/fraction	SIB squamous cell carcinoma of the right tonsil plan with 66, 59.4, and 54 Gy treatment volumes
6	HN	69.96 Gy 2.12 Gy/fraction	SIB squamous cell carcinoma of the left maxillary sinus with 66, 59.4, and 54 Gy treatment volumes, eyes receiving ALARA dose. Two plans, one optimized with 500 iterations (6.a) and one with 2000 iterations (6.b).

HN: head‐and‐neck; GYN: gynecological.

### 2.A MF determination from LOT distribution

**Figure 2:**
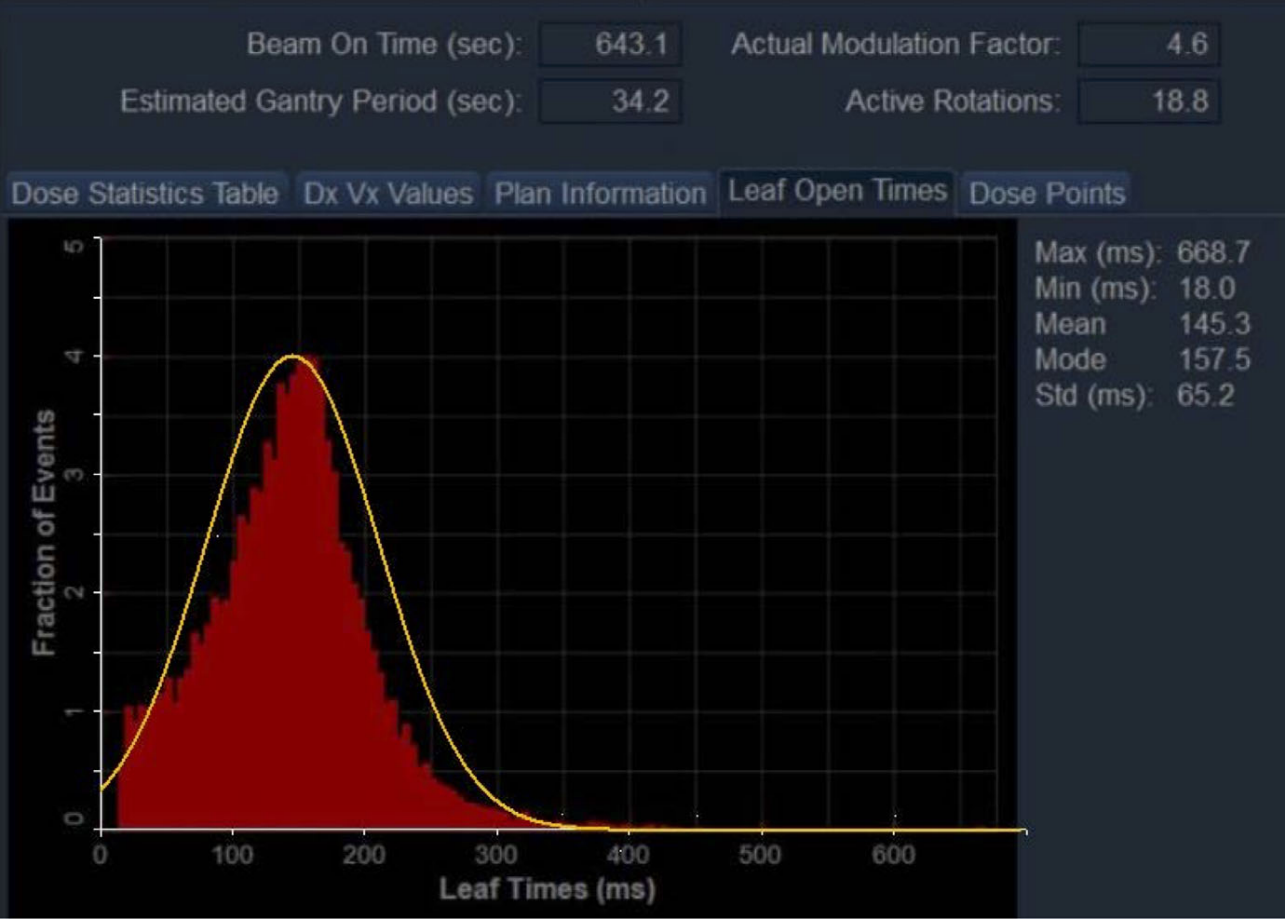
Approximation of unimodal leaf‐open time (LOT) distribution by an un‐normalized Gaussian having a mean value of 145.3 ms and a standard deviation of 65.2 ms of the LOT distribution.

In the planning system the LOT histogram is described by three parameters; its mean, mode, and standard deviation. Figure 2 shows an example of a LOT histogram that is unimodal and approximately bell‐shaped. For many of the treatment plans we have looked at we have found such a LOT histogram. There are however, instances where this is not the case and we have included a number of examples of LOT distributions in this paper that are not unimodal (cf. Figs. 5 and 6 below). However, as long as an MF ≤ 5 suffices for plan generation, one feature that remains preserved for all LOT histograms is that they exhibit an exponential fall‐off for large leaf open times. If an MF ≥ 5 is required then this leads to an accumulation of fractional events in the highest leaf open time bin of the LOT histogram spoiling the exponential fall‐off.

One can expect this shape of the LOT histogram if one assumes that the leaf open times of each of the 64 MLC leaves comprising the binary Tomotherapy MLC are arbitrarily distributed for a given treatment plan, that is, one assumes a plan specific but arbitrary leaf open time distribution for each individual MLC leaf. Moreover, let us assume that for a given plan the leaf open times for all open MLC leaves are distributed independently. This is a very strong assumption that in reality is almost certainly violated to some extent since within a given projection the leaf open times for neighboring MLC leaves are very likely correlated. However, for the sake of argument let us for assume that this assumption holds. Then let k ≤ 64 denote the number of MLC leaves that are opened for a given plan and let N_LOT_ denote the total number of MLC leaf open times making up the LOT histogram for a given plan (total population of LOT). We now select N_LOT_/k independent samples consisting of k leaf open times, one leaf open time for each open MLC leaf, from the total population of leaf open times. For each of the N_LOT_/k samples we determine its sample mean $\mu_{i}^{\mathrm{LOT}};i\in \left\{1,\ldots ,N_{\mathrm{LOT}}/k\right\}$; the central limit theorem states that no matter how the LOT values in the population are distributed, the distribution of these $\mu_{i}^{\mathrm{LOT}};i\in \left\{1,\ldots ,N_{\mathrm{LOT}}/k\right\}$, will approximate a Gaussian distribution if the sample size is large enough. Assuming that each of the k MLC leafs is at least opened once in at least one of the projections, the minimum value for N_LOT_/k = 51, which definitely meets the requirement of our sample being large enough. A moment's thought shows that if one draws the histogram showing the distribution of these N_LOT_/k sample means it will look like the one shown in Fig. 2. In fact, in what follows we interpret the leaf times given in the LOT histogram as $\mu_{i}^{\mathrm{LOT}}$values. We have made this explicit in Fig. 2 by fitting an un‐normalized Gaussian to the shown LOT histogram whose mean value is equal to that of the LOT histogram. As pointed out above, in general one has to expect deviations from this form of the LOT histogram since neighboring MLC leaf open times are very likely correlated to each other within any projection violating our assumption of independent leaf open times. The extent to which this assumption is violated will lead to a departure from the unimodal appearance of the LOT histogram. The method of picking a cutoff leaf open time discussed below however, remains valid as long as the resulting LOT histogram exhibits an exponential fall‐off for large leaf open times.

In our approximation we have set the mean of our un‐normalized Gaussian equal to the mean of the LOT distribution and have taken the standard deviation of LOT distribution as its standard deviation. Using this approximation to the LOT distribution, as one is justified to do by the central limit theorem, one can easily calculate a cutoff LOT_max_ value that eliminates the upper tail of LOTs using the following equation,

(4) $$\mathrm{LO}T_{\mathrm{Max}}\left(z_{\mathrm{critical}}\right)=\mathrm{LO}T_{\mathrm{Mean},\mathrm{final}}+z_{\mathrm{critical}}\times \mathrm{LO}T_{\mathrm{Std},\mathrm{final}},$$

where LOT_Mean,final_ is the mean of the LOT distribution after final dose calculation, and LOT_Std,final_ is the standard deviation after final dose calculation, and *z*_*critical*_ is the critical z‐value corresponding to percentage of upper LOTs that would be filtered from a true Gaussian distribution (Table 2). This LOT_max_ value was then used in Eq. (1) to determine the MF for subsequent “reduced‐MF plan” calculations. However, to maintain LOT_Max_ cutoff value, and therefore the intended reduced delivery time, the mean of the optimization LOT distribution was used to calculate MF, that is,

(5) $$MF\left(z\right)=\frac{\mathrm{LO}T_{\mathrm{Max}}\left(z_{\mathrm{critical}}\right)}{\mathrm{LO}T_{\mathrm{Mean},\mathrm{opt}}}.$$

**Table 2:** Z‐score vs percent of highest leaf‐open times (LOTs) restricted to new LOT_max_

*z*_*critical*_	2.33	1.96	1.65	1.28	0.86
Cutoff percent	1%	2.5%	5%	10%	20%

This is done because the mean of the LOT distribution shifts as a result of inclusion of the bins with less than 20 ms in determining the mean of the distribution. While the final deliverable MF is less than or equal to the planning MF because of exclusion of bins with less than 20 ms, but both share the same LOT_max_ value, which is what we are adjusting to find an efficient “beam‐on” delivery time. This lower leaf open time cutoff is due to the 20‐ms transit time of MLC leaf opening and closing.

### 2.B Progressive MF reduction

A reference plan was generated with highest MF allowed (MF = 5) using 300 iterations for the simple prostate plans and 500 iterations for more complex simultaneous integrated boost (SIB) plans with the exception of one HN plan (6.b) that was optimized with 2000 iterations to compare with the same plan optimized with 500 iterations (6.a). Optimization was done at medium resolution (1.96 mm × 2.5 mm × 1.96 mm) and final dose calculation was done at high resolution (0.98 mm × 2.5 mm × 0.98 mm). LOT_Mean,final_; LOT_Mean,opt_; and LOT_Std,final_ from the reference plan LOT distribution were used to generate five levels of modulation (Table 2) for subsequent reduced‐MF plan calculations starting with MF(*z*_*critical*_ = 2.33) and stepping down to MF(*z*_*critical*_ = 0.86) with 50 iterations per step.

After the reference plan was generated dose‐volume region of interests (ROIs) were established in the Precision Dx Vx table (Fig. 1) for comparison with reduced‐MF plans as they were calculated. For each target volume the dose‐volume ROIs were D_2%_ and D_98%_. For each critical structure volume the dose‐volume ROIs were D_0.03 cc_ and any dose volume histogram (DVH) percent volume value used as constraints for optimization. ROIs were categorized for the purpose of plan comparison between reference and reduced‐MF plans. Table 3 lists the three different dose‐volume ROI categories and the dose‐difference tolerances relative to prescription dose. The first type is associated with PTVs and partial PTV volumes used for optimization. The second type is associated with organ at risk (OAR) dose‐volume and point dose constraints that are critical to achieving clinical planning goals. The third type is associated with dose‐shaping and noncritical OAR constraints meant to achieve lowest allowed doses during optimization such as ring structures.

**Table 3:** Plan difference criteria for region of interest (ROI) constraints

ROI type	Max difference	Dose‐volumes structures
PTV	1% of Rx Dose	PTV volumes and partial volumes for optimization
OAR	2% of Rx Dose	OAR volumes and partial volumes (e.g., residual parotid region) critical to clinical objectives
Tuning	3% of Rx Dose	Rings, dose‐shaping volumes (e.g., post neck region), and noncritical OAR ROI constraints

OAR: organ at risks; PTV: planning target volumes.

Each reduction in MF was followed with an additional 50 iterations of optimization to allow redistribution of energy fluence among the beamlets affected [e.g., a total of 550 iterations were done for a prostate plan with a final modulation of MF(*z*_*critical*_ = 0.86)]. The Dx Vx table was reviewed following each reduction of MF and subsequent additional 50 iterations. If ROIs were found to have a dose difference with the reference plan ROIs greater than the tolerance level for its category, the plan was recalculated from the last successful *z*_*critical*_ level with adjustments to weighting and penalty factors to the ROIs out of tolerance.

## 3 RESULTS

Table 4 lists the reduced MF as a function of the *z*_*critical*_ score using Eq. (5) for each reference plan and associated LOT standard deviation along with the estimated time of delivery using Eq. (3); the entry in bold is the lowest MF that maintained ROI dose‐difference tolerances with the reference plan. Also listed in Table 4 are the fraction of LOT events in the LOT_max_ bin. These values are slightly higher than those listed in Table 2, especially for more complex plans, but do seem to indicate that the central limit theorem is valid in describing the LOT distribution.

**Table 4:** Modulation factor (MF) as a function of critical‐z score determined from reference (ref) plan leaf‐open time (LOT) distribution and its standard deviation (std). Also listed are the estimated time of delivery (values are shown in italics) and the fraction of LOT events that occupy the LOT_max_ bin. The entries shown in bold for each reference plan was the plan with lowest MF with dose‐volume region of interests (ROIs) within dose‐difference tolerances

Ref Plan	LOT Std	MF(*z*_*critical*_ * *= 2.33) Delivery time % LOT_max_	MF(*z*_*critical*_ * *= 1.96) Delivery time % LOT_max_	MF(*z*_*critical*_ * *= 1.65) Delivery time % LOT_max_	MF(*z*_*critical*_ * *= 1.28) Delivery time % LOT_max_	MF(*z*_*critical*_ * *= 0.86) Delivery time % LOT_max_
1.a	81.2 s	*2.17* *167.8 s* *2.3%*	*1.99* *154.2 s* *3.4%*	*1.84* *142.6 s* *6.0%*	**1.67** **129.1 s** **11.9%**	*1.46* *112.9 s* *22.4%*
1.b	114.1 s	*2.67* *208.4 s* *3.3%*	*2.42* *189.2 s* *5.6%*	**2.22** **172.9 s** **7.6%**	*1.97* *154.0 s* *13.9%*	*1.68* *131.2 s* *26.9%*
2.a	75.0 s	*2.22* *178.8 s* *3.7%*	*2.04* *164.5 s* *5.3%*	**1.89** **152.4 s** **8.1%**	*1.71* *138.3 s* *12.6%*	*1.50* *121.3 s* *24.9%*
2.b	88.9 s	*2.37* *190.3 s* *3.8%*	**2.17** **174.4 s** **5.6%**	*2.00* *160.8 s* *8.1%*	*1.81* *145.1 s* *13.6%*	*1.57* *126.2 s* *25.2%*
3	89.0 s	*2.82* *3* *52.4 s* *04.0%*	*2.55* *319.1 s* *5.4%*	**2.33** **290.8 s** **8.8%**	*2.06* *258.0 s* *12.1%*	*1.75* *218.5 s* *22%*
4	122.0 s	*3.47* *400.4 s* *4.3%*	*3.12* *360.4 s* *7.9%*	*2.83* *326.6 s* *10.6%*	**2.49** **287.3 s** **14.8%**	*2.08* *240.0 s* *24.0%*
5	86.9 s	*2.79* *364.0 s* *3.6%*	*2.53* *330.4 s* *6.3%*	**2.31** **301.8 s** **10.2%**	*2.06* *268.6 s* *15.8%*	*1.75* *228.6 s* *27.3%*
6.a	169.4 s	**3.58** **650.7 s** **5.2%**	*3.21* *582.9 s* *7.6%*	*2.89* *525.2 s* *11.1%*	*2.52* *458.4 s* *16.0%*	*2.08* *377.8 s* *25.8%*
6.b	220.8 s	*4.61* *840.9 s* *6.4%*	**4.13** **752.6 s** **7.8%**	*3.72* *677.4 s* *10.0%*	*3.24* *590.3 s* *13.4%*	*2.66* *485.2 s* *20.7%*

**Figure 3:**
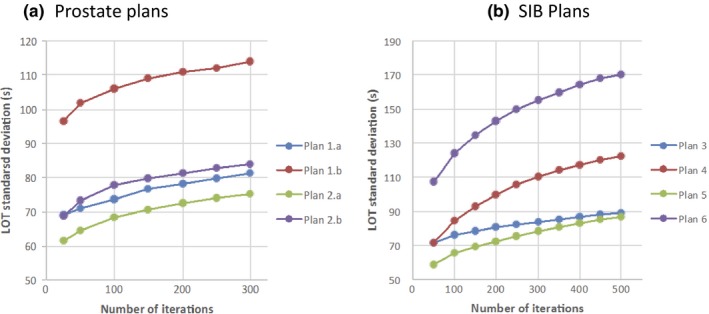
Leaf‐open time (LOT) standard deviation (s) vs number of optimization iterations for prostate plans (a) and SIB plans (b).

Figure 3 shows the standard deviation of the LOT distribution vs. iteration for the six treatment cases. There is rapid expansion of the LOT distribution within the first 100–200 iterations followed by a gradual increase. The reason the number of iterations chosen for the prostate and SIB plans was 300 and 500, respectively, was (a) that in our experience these values lead to convergence to a good plan and (b) to insure that the LOT distribution had sufficiently settled into a linear increase in standard deviation with number of optimization iterations. Oliver et al also found convergence with phantom plans using 250 iterations.^21^

### 3.A Prostate plans

**Figure 4:**
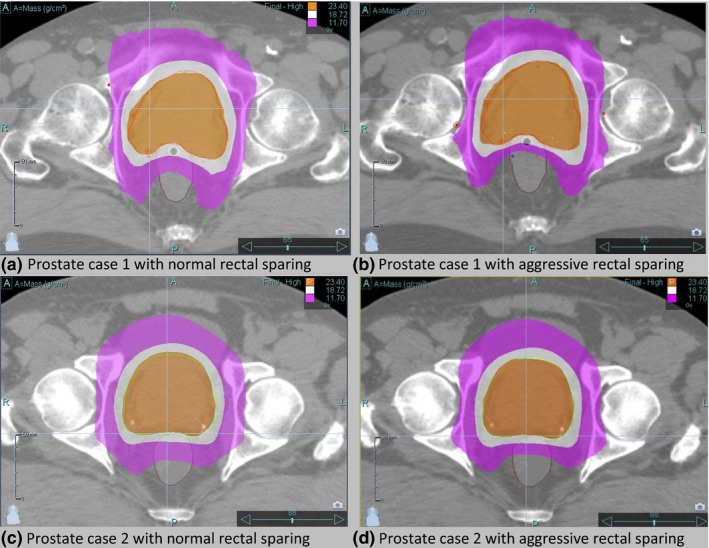
Prostate‐only boost dose distributions with shaded isodose lines of 100% (orange), 80% (white), and 50% (purple) for case 1 (a,b) and case 2 (c,d) with normal rectal sparing (a,d) and aggressive rectal sparing (b,d).

**Figure 5:**
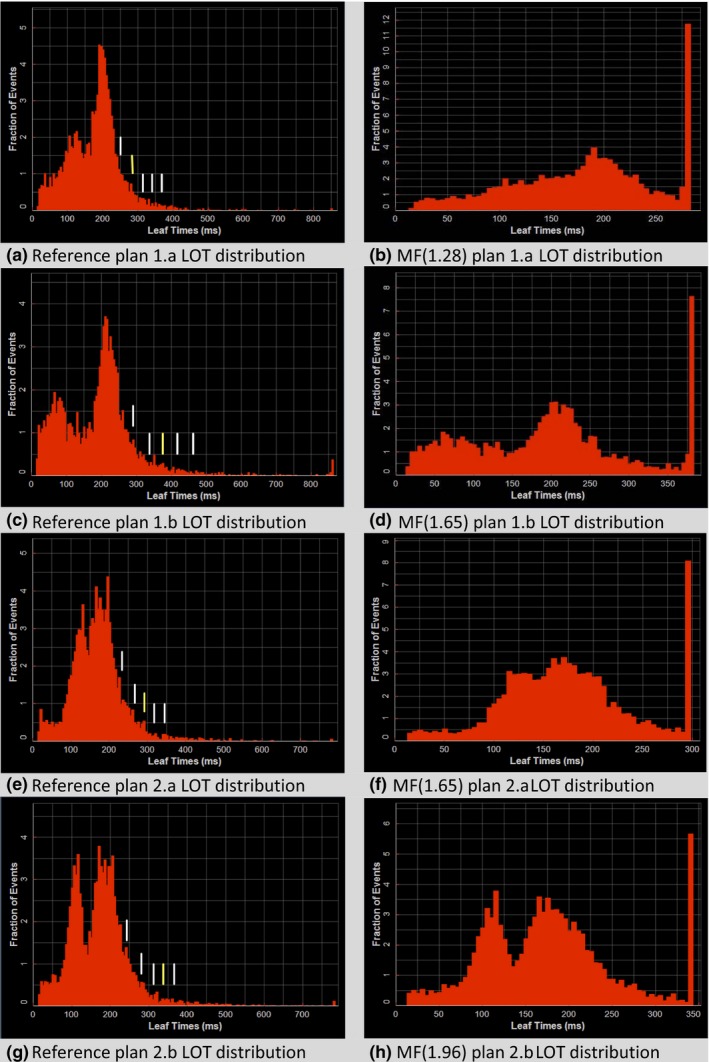
Prostate plan final dose calculation leaf‐open time (LOT) distributions for case 1 (a–d) and case 2(e–h). The yellow vertical bar shown in each of the left panels corresponds to the chosen *z*_*critical*_ value for that LOT distribution. The LOT distribution resulting using the MF determined from this *z*_*critical*_ value is shown in the corresponding right panel.

Figure 4 shows dose distributions for the plans with two different levels of rectal sparing for the two prostate plans. Figures 4(a) and 4(c) show typical rectal sparing dose fall‐off with the 50% isodose line running through the middle of the rectum while Figs. 4(b) and 4(d) show atypical dose sparing that might be done with symptomatic patients and/or rectal balloon fixation. Figure 5 shows the LOT for the reference plan and the best reduced‐MF plan for each of the prostate plans. Also shown in the reference plan LOT distribution are the cutoff LOT_max_ values determined from Eq. (4). The lower LOTs become more pronounced for plans with aggressive rectal sparing to the point where the LOT distribution appears to be bimodal. The increase in LOT_Std,final_ in these plans results in generated reduced‐MFs that increase as should be expected for pushing OAR sparing. The z‐critical value where the best reduced‐MF plan was found was slightly different between the two cases and their associated plans even though the same jaw width, pitch, and optimization importance and penalty factors were used. This could be attributed to differences in the individual geometry presented by the PTV and critical structure volumes.

### 3.B SIB plans

**Figure 6:**
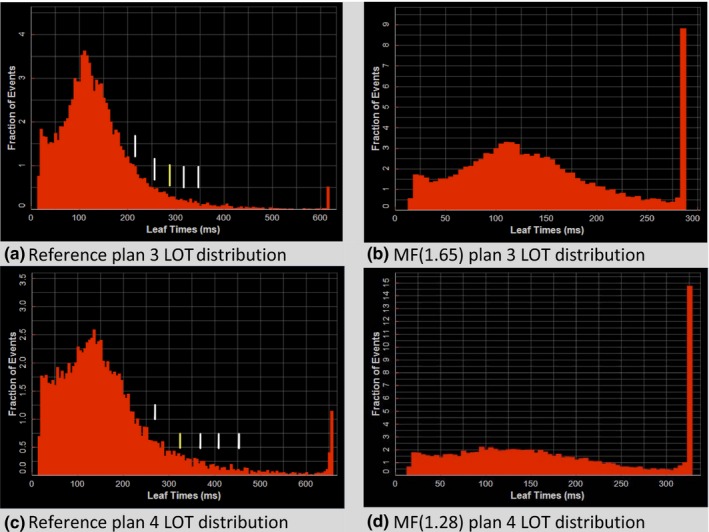
Gynecological simultaneous integrated boost plan final dose calculation leaf‐open time (LOT) distributions for case 3 (a,b) and case 4 (c,d). The yellow vertical bar shown in each of the left panels corresponds to the chosen *z*_*critical*_ value for that LOT distribution. The LOT distribution resulting using the MF determined from this *z*_*critical*_ value is shown in the corresponding right panel.

**Figure 7:**
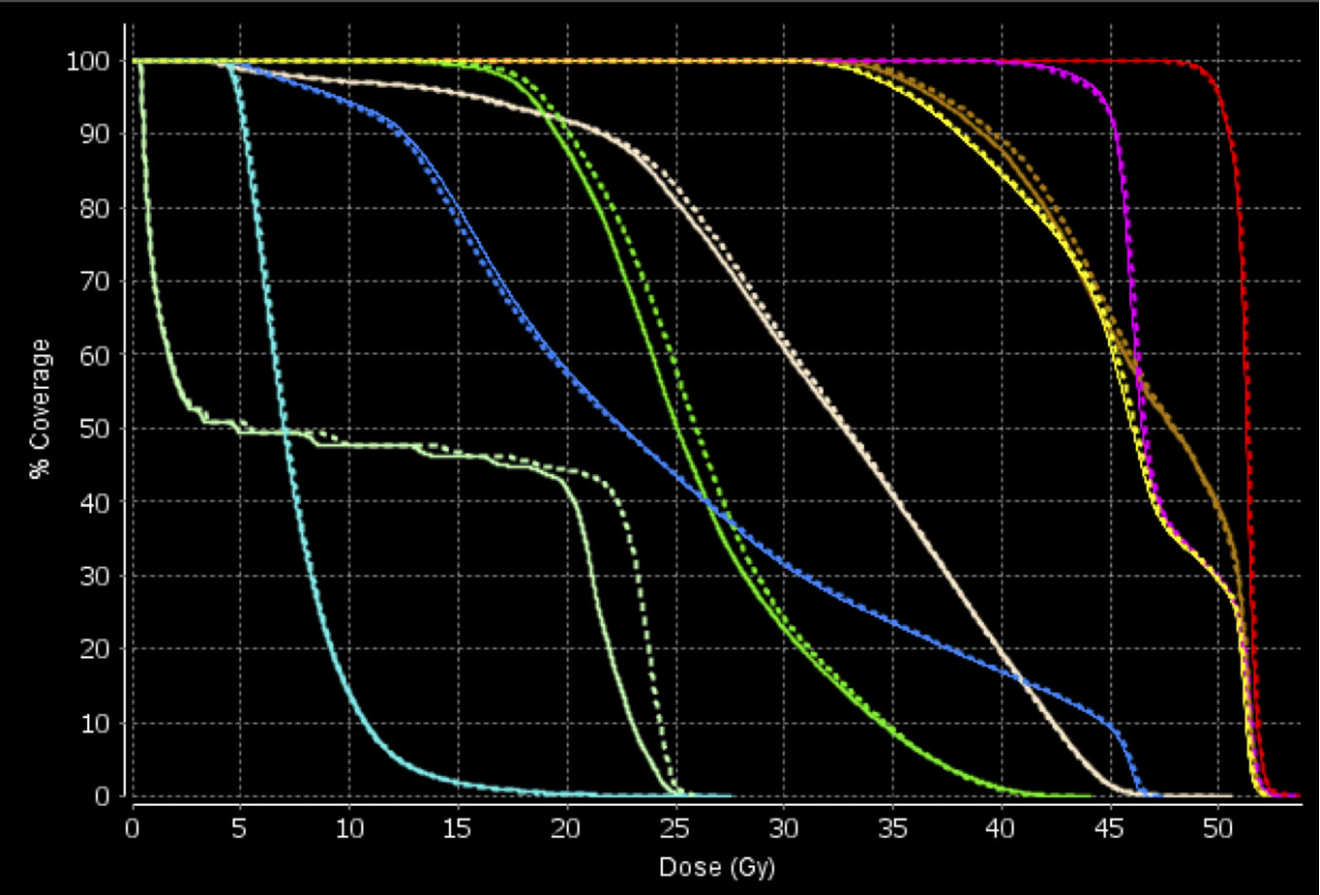
Dose volume histogram comparison between reference plan (solid lines) and MF(*z*_*critical*_ = 1.28) plan (dashed lines) for case 4. Structures shown are PTV50 Gy (red), PTV45 Gy (magenta), bladder (yellow), rectum (brown), bowel (blue), combined femoral heads (green), combined kidneys (cyan), spinal cord (light green), and dose‐shaping ring (almond).

Figure 6 shows reference and reduced‐MF plan LOTs for each of the GYN plans. The reduced MF for plan 3 is 2.33. The LOT standard deviation is larger for plan 4 because the 45 Gy PTV is larger and there is considerable dose sparing for the kidneys due to a pre‐existing condition. As a result, the reduced MF for plan 4 is 2.49; what is remarkable is that lowest LOT‐generated MF with dose‐volume ROIs within dose‐difference tolerances is at a *z*_*critical*_ = 1.28, resulting in the dominant LOT_max_ bin in the LOT distribution. Figure 7 shows the DVH comparison of the MF(*z*_*critical*_ = 1.28) plan with the reference plan calculated at MF = 5.0. Very good agreement is seen with the PTVs and critical structures shown. The largest difference is 2 Gy seen with the spinal cord V30 but this was not controlled by an optimization dose‐volume critical structure constraint, only the maximum dose was.

**Figure 8:**
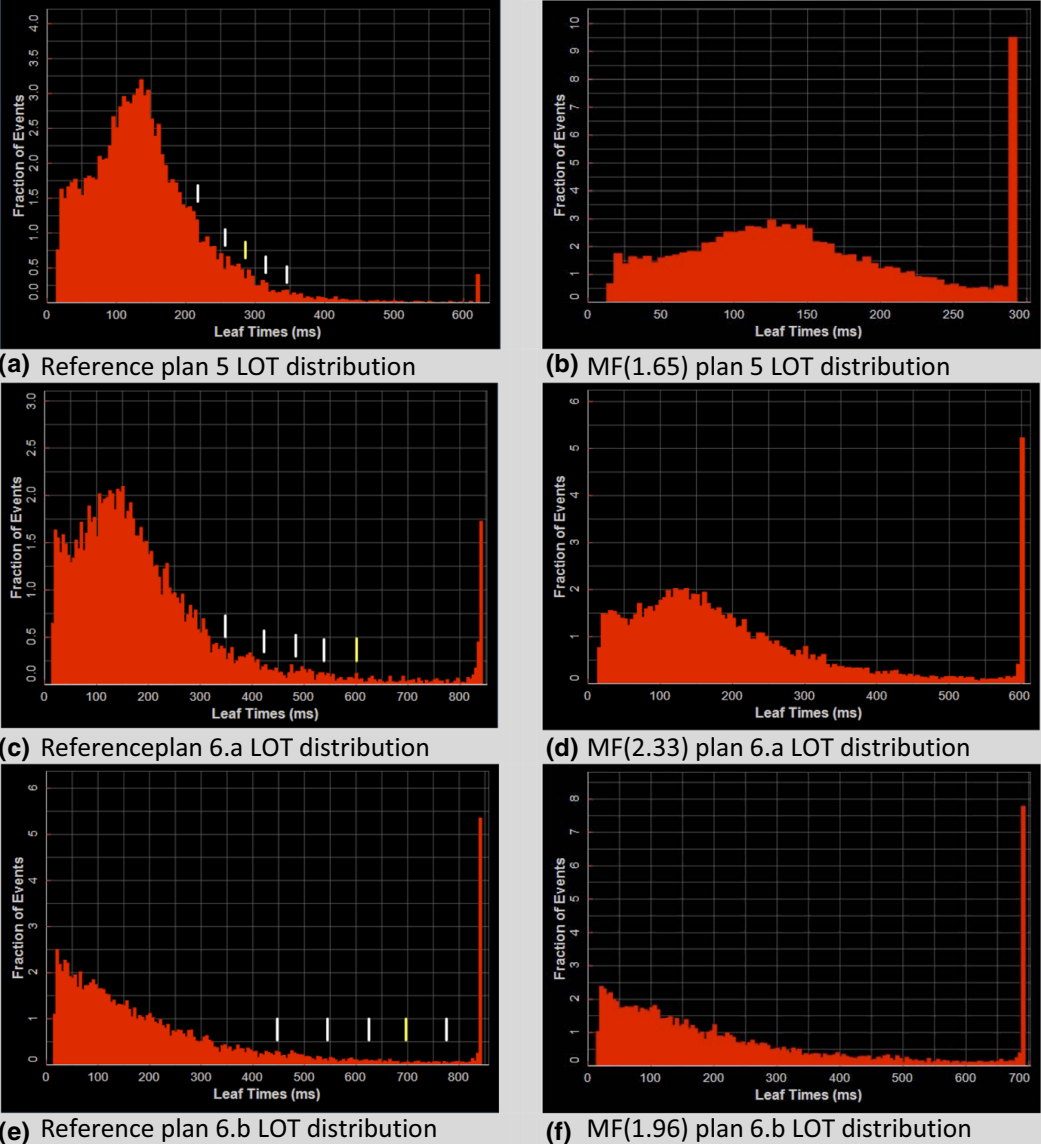
Head‐and‐neck simultaneous integrated boost plan final dose calculation leaf‐open time (LOT) distributions for case 5 (a,b) and case 6 (c–f). The yellow vertical bar shown in each of the left panels corresponds to the chosen *z*_*critical*_ value for that LOT distribution. The LOT distribution resulting using the MF determined from this *z*_*critical*_ value is shown in the corresponding right panel.

**Figure 9:**
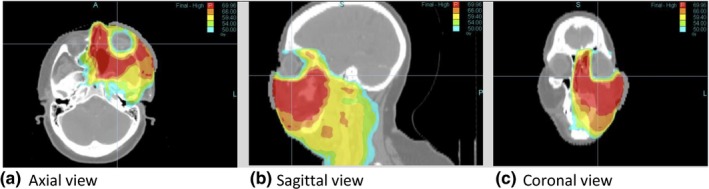
Dose distribution for case 6 showing complexity of plan with isodose wash levels of 69.96 (red), 66 (orange), 59.4 (yellow), 54 (green), and 50 Gy (light blue).

Figure 8 shows the reference and reduced‐MF plan LOTs for each of the HN plans. For case 5 the reduced MF is 2.31. For case 6.a, however, a reduced‐MF plan that had ROI dose differences within tolerances could not be achieved below a *z*_*critical*_ score of 2.33. This plan was particularly complex because of proximity of PTV to the left orbit as shown in Fig. 9 and the desire to limit the dose as much as possible to the right eye. The resulting reduced MF for case 6.a was 3.58. Plan 6.b was the same as plan 6.a with number of optimization iterations taken to 2000. The LOT distribution for reference plan 6.b shown in Fig. 8(e) clearly shows how spread out the LOT distribution becomes compared to the 500 iteration plan [Fig. 8(c)]. While there was marginal improvement with reference plan 6.b compared to 6.a (e.g., PTV69.96 D_2%_ was 1 Gy cooler), the resulting reduced MF value was 4.13 and the difference in delivery time between the two reduced‐MF plans was approximately 100 s.

**Figure 10:**
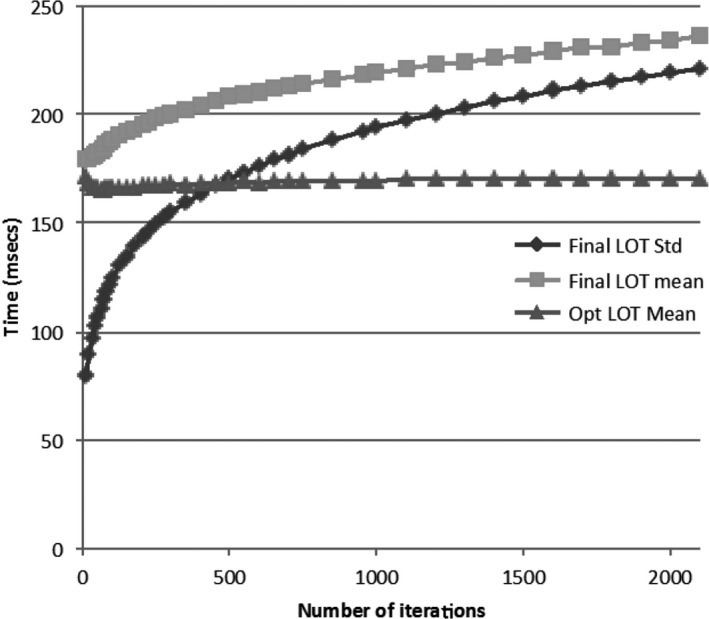
Plot of leaf‐open time (LOT) standard deviation, final mean, and opt mean for plan 6.b vs number of optimization iterations.

Figure 10 shows the increase in LOT standard deviation and mean with number of optimization iterations for plan 6.b which was taken to 2000 iterations. It shows that the LOT_Std, final_ and LOT_Mean, final_ grows steadily after 500 iterations while the LOT_Mean,opt_ remains relatively constant. The difference between LOT_Mean,final_ and LOT_Mean,opt_ is due to the continual buildup in the <20 ms LOT bins during optimization that are eliminated in the final delivery plan.

## 4 DISCUSSION AND CONCLUSION

Using the central limit theorem, we have derived a methodology that allows a planner to arrive at a reduced MF from the LOT distribution of a high MF treatment plan. A scheme for finding a reduced MF from a set of MFs that results in a plan balanced in both dosimetric quality and treatment delivery efficiency has also been presented. The combination of the two should allow a planner to efficiently determine a suitable helical IMRT plan MF for most clinical situations and planning expectations.

It should be emphasized that the method presented in this paper demonstrates an alternate approach in which an MF that balances both treatment delivery time and dosimetric quality is determined after initially planning with the highest available MF. A good quality and efficient plan can certainly be achieved with a MF or range of values established by experience and clinical protocols. Most of the MFs determined by the method presented in this paper fall within the range of published values. The MFs for the prostate cases ranged from 1.67 to 2.22 compared to the range 1.8 to 3.5 of published values and are within the 2.2 upper limit in the study by Shimizu et al.^14^ The MF for GYN case 3 and 4 was 2.33 and 2.49, respectively, compared to the published ranges of 3.0 to 4.0.^12^, ^13^ This would suggest that some of the GYN cases in these studies could have benefitted from our technique. The MF for HN case 5 and 6.a was 2.31 and 3.58, respectively, which also fell within the published ranges of 2.0 to 3.5.^7^, ^8^, ^9^, ^10^, ^11^ However, a MF of 3.58 for case 6.a falls outside Shimizu et al's upper limit value of 2.6 for HN planning.^14^ Case 6.a was a particularly complex HN plan that resulted in a high MF that was beyond the two standard deviations of limit in Shimizu's distribution of HN MFs. This also underscores Skórska et al.'s conclusion that complex HN planning should start with MF = 5 with MF reduction during optimization until a suitable balance between plan quality and delivery efficiency is achieved.^9^ The methodology presented in this paper provides the user with a stepwise MF reduction scheme that should achieve such a balance within a few steps.

Plan evaluation and acceptance criteria can vary depending on individual clinical expectations and dosimetric quality trade‐offs. The criteria for plan comparison used in this study were chosen to find the best MF within a range of MFs using an established set of rules and are not meant to be standard practice for plan evaluation. The logic was to allow dose‐shaping constraints to be more flexible than critical OAR constraints while maintaining the original PTV dose homogeneity as much possible. Rarely did dose‐shaping ROIs exceed the 3% dose‐difference tolerance. Given the initial importance and penalty values of the volumes the ROIs were associated with were much lower than the PTV and critical OAR volumes, small adjustments to the values during MF‐reduction optimization steps were usually sufficient in keeping the dose‐shaping ROIs within tolerance. Finding the best MF factor was usually a planning balance between PTV ROIs and one or two critical OAR ROIs. The Rx PTV that is normalized at a specified dose‐volume value upon final dose calculation is the most important because of global shifting in all DVHs.

Anatomy and OAR sparing affects the LOT distribution as evident in the plans presented. For example, aggressive sparing of the rectum results in LOT distributions with larger standard deviation and higher MFs. This is intuitively correct as larger MFs are required for complex planning and dose shaping. The MFs derived from the LOT distribution fall within the ranges reported by other investigators. The exceptional HN case involving the left orbit shows that sometimes a high MF is warranted. Hence, this method is ideal for situations where a good MF is not known beforehand and having a small range of MFs to generate a good plan allows one to arrive at an efficient delivery time with acceptable dosimetric and plan quality trade‐offs.

Using the LOT distribution to determine MF is independent of jaw width and pitch. However, using tight pitch values will limit how much the maximum LOT time can be reduced as rotation period has a minimum value of 11.8 s. For example, in the first prostate case presented the mean LOT was 182 ms. Halving the pitch factor from 0.430 to 0.215 effectively halves the mean LOT value. The lowest allowed LOT_max_ time is 235 ms which would limit the lowest possible MF to approximately 2.58. Plans presented in this study used pitch factors above 0.4 given the range of dose per fraction for plan was between 1.8 and 2.12 Gy.

Negative LOTs are forced to “zero value” during optimization which can effectively cutoff the lower part of the LOT distribution [e.g., the GYN reference plan 3 LOT distribution shown in Fig. 6(a)] thereby skewing the LOT distribution of the reference plan from a normal distribution. The upper tail of the LOT distribution is mostly unaffected unless the plan requires an MF that lies above the currently possible maximum MF that can be set in the planning system, as is evident in the buildup of LOT_max_ in the more complex SIB plans presented [e.g., 1.2% for the LOT_max_ bin in GYN reference plan 4 shown in Fig. 6(c)]. Aggressive OAR sparing and beamlet blocking can skew LOT distributions also, as evident in the prostate plans where aggressive sparing of the rectum produced bimodal LOT distributions [Figs. 5(c) and 5(g)]. However, even in these cases our method generating MFs from the LOT distribution shows utility even though the distribution looks far from a standard Gaussian distribution.

The fraction of LOT events in the maximum bin ranged between 5% and 15% with an average value of 8.9% for the final efficient MFs determined in this study. This would suggest an expected range for the fraction of LOT events in the maximum bin for plan evaluation of treatment delivery efficiency. Further studies with a more comprehensive dataset would be needed to validate this approach.

The LOT distribution expands with number of optimization iterations as seen in Figs. 3 and 10; this has an impact on the MF derived from the LOT distribution. One reason to limit each step in the progressive MF reduction scheme to 50 iterations was to minimize the impact of an ever‐widening LOT distribution when trying to improve differences with adjustments to ROI weighting and penalty values. It was found from experience that 50 iterations per step MF reduction was sufficient in allowing the LOT distribution to adjust. The other reason was to present an efficient scheme for finding good MF that a planner could utilize. We did not evaluate different MF reduction schemes since the focus of this study was on presenting a proof of concept that a good MF can be determined from the LOT distribution based on a general argument involving the central limit theorem. Further investigation of different MF reduction schema might produce interesting insights and more efficient planning schemes.

## CONFLICTS OF INTEREST

The authors declare no conflicts of interest.
